# Incidence and influence of prosthesis-patient mismatch after reoperative aortic valve replacement: a retrospective single-center study

**DOI:** 10.1186/s13019-020-01094-2

**Published:** 2020-03-30

**Authors:** Hideki Tsubota, Genichi Sakaguchi, Akira Marui

**Affiliations:** Department of Cardiovascular Surgery, Kokura Memorial Hospital, Asano 3-2-1, Kokura Kita-ku, Kitakyushu, Fukuoka, 802-8555 Japan

**Keywords:** Aortic valve replacement, Heart valve prosthesis, Reoperation

## Abstract

**Background:**

Reoperative aortic valve replacement (AVR) is associated with increased mortality compared with initial surgery, and a smaller valve might be implanted during repeat AVR (re-AVR; AVR after prior AVR). We describe the clinical outcomes and incidence of prosthesis-patient mismatches (PPM) after reoperative AVR.

**Methods:**

Among 113 patients who underwent reoperative AVR between 2007 and 2018, 44 underwent re-AVR and 69 underwent a first replacement of a diseased natural valve after any cardiac surgery except AVR (primary AVR). We then compared early and late outcomes, the impact of re-AVR on the effective orifice areas (EOA), and the incidence and influence of PPM on reoperative AVR.

**Results:**

Hospital mortality was 2.7%, and the overall 1-, 3-, and 5-year survival rates were 95, 91 and 86%, respectively. The reference EOA of the newly implanted valve was smaller than that of the previous valve (1.4 ± 0.3 vs. 1.6 ± 0.3 cm^2^, *p* < 0.01). The mean pressure gradient was greater (15.2 ± 6.4 vs. 12.7 ± 6.2 mmHg, *p* = 0.04) and indexed EOA was smaller (0.92 ± 0.26 vs. 1.06 ± 0.36 cm^2^/m^2^, *p* = 0.04) during re-AVR than primary AVR, whereas the incidence of PPM was similar (38.7% vs. 34.8%, *p* = 0.87) between the groups.

**Conclusions:**

The clinical outcomes of reoperative AVR were acceptable. Although the reference EOA of new implanted valves was smaller than that of previous valves, re-AVR did not increase the incidence of PPM. These findings might serve as a guide for future decisions regarding the surgical approach to treating degenerated prosthetic valves.

## Background

The prevalence of reoperative aortic valve replacement (AVR) after prior cardiac surgery is increasing due to increased survival rates and lifespans. In addition, a trend towards implantation with bioprostheses means that more patients will need to undergo repeat AVR (re-AVR; AVR after previous prosthetic AVR) due to structural valve deterioration (SVD).

Reoperative cardiac surgery is considered to be high-risk because of the technical difficulties involved in operating within an adhered field, which is associated with risk of heart injury while opening the chest and excessive blood loss. Indeed, the in-hospital mortality after reoperative AVR is 7.1% compared with only 1.9% after initial AVR in Japan [1]. In addition, patients with a previous AVR who undergo reoperation are regarded as being at higher risk of a prosthesis-patient mismatch (PPM), due to annular fibrosis and restriction after prosthesis extraction; however, no evidence has been published on the incidence of PPM after re-AVR. Meanwhile, transcatheter aortic valve-in-valve (VIV) implantation has emerged as a less invasive option for destroyed bioprostheses with SVD, and the early outcomes of transcatheter aortic VIV implantation are favorable [2–4], although the incidence of PPM is high [2].

The clinical outcomes and incidence of PPM for re-AVR should be clarified to understand the indications and limitations of re-AVR and VIV. This study aimed to determine the early and late outcomes of reoperative AVR, and incidence and influence of PPM after reoperative AVR.

## Methods

### Study design and patient population

The Institutional Review Board at Kokura Memorial Hospital approved this study and waived the need for patient consent because of its retrospective single-center design. The characteristics of the patients, type of procedure, postoperative outcomes and follow-up data were retrospectively retrieved from institutional databases.

Between November 2007 and December 2018, 113 patients underwent reoperative AVR (AVR after any type of previous cardiac surgery including AVR). Among these, 44 underwent re-AVR and 69 underwent primary AVR (after any previous cardiac surgery except AVR). All surgeries proceeded at the Division of Cardiovascular Surgery and Cardiology, Kokura Memorial Hospital, Fukuoka, Japan.

### AVR procedure

Reoperations were proceeded via a standard median repeated sternotomy followed by dissection and identification of the cardiac structures. The ascending aorta and the subclavian artery or femoral artery were cannulated. A venous cannula was inserted into the right atrium or femoral vein. The myocardium was protected by antegrade cold crystalloid cardioplegia without topical cooling, injected into the aortic root or directly into the native coronary and graft ostia. Patent bypass grafts were dissected and clamped during aortic cross-clamping. Preexisting prosthetic valves were explanted by cutting all tied sutures and completely excluding the cuff cloth and pledgets on aortic annula. Further, native aortic annula were exposed and the pannus on annula were removed. The annular size was measured using a sizer for each valve type. The type of prosthesis was selected according to the profiles of individual patients. The aortic annulus or root was not enlarged in this series.

### Prosthesis-patient mismatch

We used postoperative echocardiography to determine the presence of PPM. We measured and indexed the effective orifice areas (EOA) of the prosthetic AV to the body surface area of each patient. The normal indexed EOA (EOAI) value is > 0.85 cm^2^/m^2^. A PPM was categorized as moderate or severe if the EOAI ranged from 0.65–0.85 cm^2^/m^2^ or was < 0.65 cm^2^/m^2^, respectively.

We also compared the reference EOA between explanted and implanted valves in the re-AVR group. The reference EOA for each given model and the prosthesis size were determined from the literature [5–12].

### Endpoints

The primary endpoint was in-hospital and late mortality rates. Other endpoints comprised perioperative complications, the incidence of PPM and the influence of PPM on late survival.

### Statistical analyses

Categorical variables were compared using Chi-squared or Fisher exact tests. Continuous variables were analyzed using Student t tests. Survival was calculated using Kaplan-Meier methods with 95% confidence intervals (CI). Statistical significance was indicated at *p* < 0.05. Survival was analyzed using the Kaplan-Meier method, and differences in survival between groups were examined using log-rank tests. All data were analyzed using SPSS v.20 statistical software (IBM SPSS Inc., Chicago, IL, USA).

## Results

### Preoperative data

Table 1 summarizes the preoperative characteristics of the patients. More patients in the re-AVR group had endocarditis, whereas most preoperative clinical variables were similar between the groups. The mean intervals between the present and previous cardiac surgeries were 11 ± 8.1 and 15.7 ± 9.8 years after re-AVR and primary AVR, respectively (*P* = 0.01). The mean follow-up duration was 4.3 ± 3.3 years.

**Table 1:** Baseline characteristics of the patients

	Total (*n* = 113)	Repeat AVR (*n* = 44)	Primary AVR (*n* = 69)	*p*
Male (%)	54 (47.8)	22 (50)	54 (78.3)	0.85
Age (years)	68.4 ± 12.8	70.4 ± 13.7	67.2 ± 12.1	0.2
Body mass index	22.5 ± 4.1	22.7 ± 4.9	22.4 ± 3.6	0.68
Body surface area	1.5 ± 0.2	1.5 ± 0.2	1.5 ± 0.2	0.81
Diabetes (%)	26 (23)	9 (20.5)	17 (24.6)	0.65
Hypertension (%)	61 (54)	26 (59.1)	35 (50.7)	0.44
Dyslipidemia (%)	38 (33.6)	14 (31.8)	24 (34.8)	0.84
Smoking history (%)	30 (26.5)	12 (27.3)	18 (26.1)	1
COPD (%)	15 (13.3)	7 (15.9)	8 (11.6)	0.58
Atrial fibrillation (%)	20 (17.7)	5 (11.4)	15 (21.7)	0.21
CVD (%)	15 (13.3)	3 (6.8)	5 (7.2)	1
Low LVEF < 40% (%)	7 (6.2)	3 (6.8)	4 (5.8)	1
Emergency (%)	3 (2.7)	2 (4.5)	1 (1.4)	0.56
Hemodialysis	10 (8.8)	5 (11.4)	5 (7.2)	0.51
Endocarditis	12 (10.6)	8 (18.2)	4 (5.8)	0.06
Aortic stenosis	70 (61.9)	23 (52.3)	47 (68.1)	0.11
Interval since last cardiac surgery (y)	13.9 ± 9.4	11 ± 8.1	15.7 ± 9.8	0.01
Reoperations				0.33
First reoperation	102 (90.3)	38 (86.4)	64 (92.8)	
Second reoperation	11 (9.7)	6 (13.6)	5 (7.2)	

*AVR* aortic valve replacement, *COPD* chronic obstructive pulmonary disease, *CVD* cerebrovascular disease, *LVEF* left ventricle ejection fraction

Table 2 summarizes the types of primary cardiac surgery and indications for the present AVR. The etiologies differed between the groups. The most common indication for re-AVR was structural valve deterioration (SVD; 52.3%), followed by pannus formation (29.5%). There were 8 (18.2%) cases of endocarditis in re-AVR patients and 4 (5.8%) in primary AVR patients. In all endocarditis patients, vegetations were on the surface of the valve, and there was no subvalvular or annular abscess; therefore, no AVR included annular reconstruction. Bioprosthetic valves were explanted from 30 (68.2%) patients, and 14 (31.8%) others had a prior mechanical valve. Primary AVR was more frequently undertaken to treat calcified aortic valves (53.6%).

**Table 2:** Previous procedures and indications for present AVR

	Total (*n* = 113)	Repeat AVR (*n* = 44)	Primary AVR (*n* = 69)	*p*
Previous procedure				
AVR	44 (38.9)	44 (100)	–	–
CABG	28 (24.8)	7 (15.9)	22 (31.9)	0.08
MVR	23 (20.4)	4 (9.1)	19 (27.5)	0.02
MVP	17 (15)	3 (6.8)	14 (20.3)	0.06
Reimplantation/Remodeling	4 (3.5)	0 (0)	4 (5.8)	0.16
AVP	4 (3.5)	0 (0)	4 (5.8)	0.16
TAR	3 (2.7)	0 (0)	3 (4.3)	0.28
Others	9 (8)	2 (4.5)	7 (10.1)	0.48
Etiology				
Calcified	37 (32.7)	–	37 (53.6)	–
Degenerative	14 (12.4)	–	14 (20.3)	–
Rheumatic	7 (6.2)	–	7 (10.1)	–
Bicuspid	6 (5.3)	–	6 (8.7)	–
IE	12 (10.6)	8 (18.2)	4 (5.8)	0.06
SVD	23 (20.4)	23 (52.3)	–	–
Pannus	13 (11.5)	13 (29.5)	–	–
TAVR valve failure	1 (0.9)	–	1 (1.4)	–

*AVP* aortic valve plasty, *AVR* aortic valve replacement, *CABG* coronary artery bypass graft, *IE* infectious endocarditis, *MVP* mitral valve plasty, *MVR* mitral valve replacement, *SVD* structural valve deterioration, *TAR* total arch replacement, *TAVR* transcatheter aortic valve replacement

### Operative data

Table 3 summarizes the operative data. Perfusion and cross-clamp durations did not differ between the groups. Almost half (47.8%) of the patients underwent AVR alone, and 52 (46%) were implanted with a mechanical valve during the present procedure.

**Table 3:** Operative data

	Total (*n* = 113)	Repeat AVR (*n* = 44)	Primary AVR (*n* = 69)	*p*
Perfusion time (min)	166.8 ± 76.2	161.3 ± 67.2	170.3 ± 81.6	0.54
Cross-clamp time (min)	121.3 ± 56.3	119.8 ± 55.5	122.2 ± 57.1	0.83
Type of procedure				
Isolated AVR	54 (47.8)	25 (56.8)	29 (42)	0.18
AVR + CABG	5 (4.4)	2 (4.5)	3 (4.3)	1
AVR + mitral	34 (30.1)	9 (20.5)	25 (36.2)	0.09
AVR + other	20 (17.7)	8 (18.2)	12 (17.4)	1
Type of prosthesis				0.12
Bioprosthesis	61 (54)	28 (63.6)	33 (47.8)	
Mechanical	52 (46)	16 (36.4)	36 (52.2)	

*AVR* aortic valve replacement, *CABG* coronary artery bypass graft

**Fig. 1:**
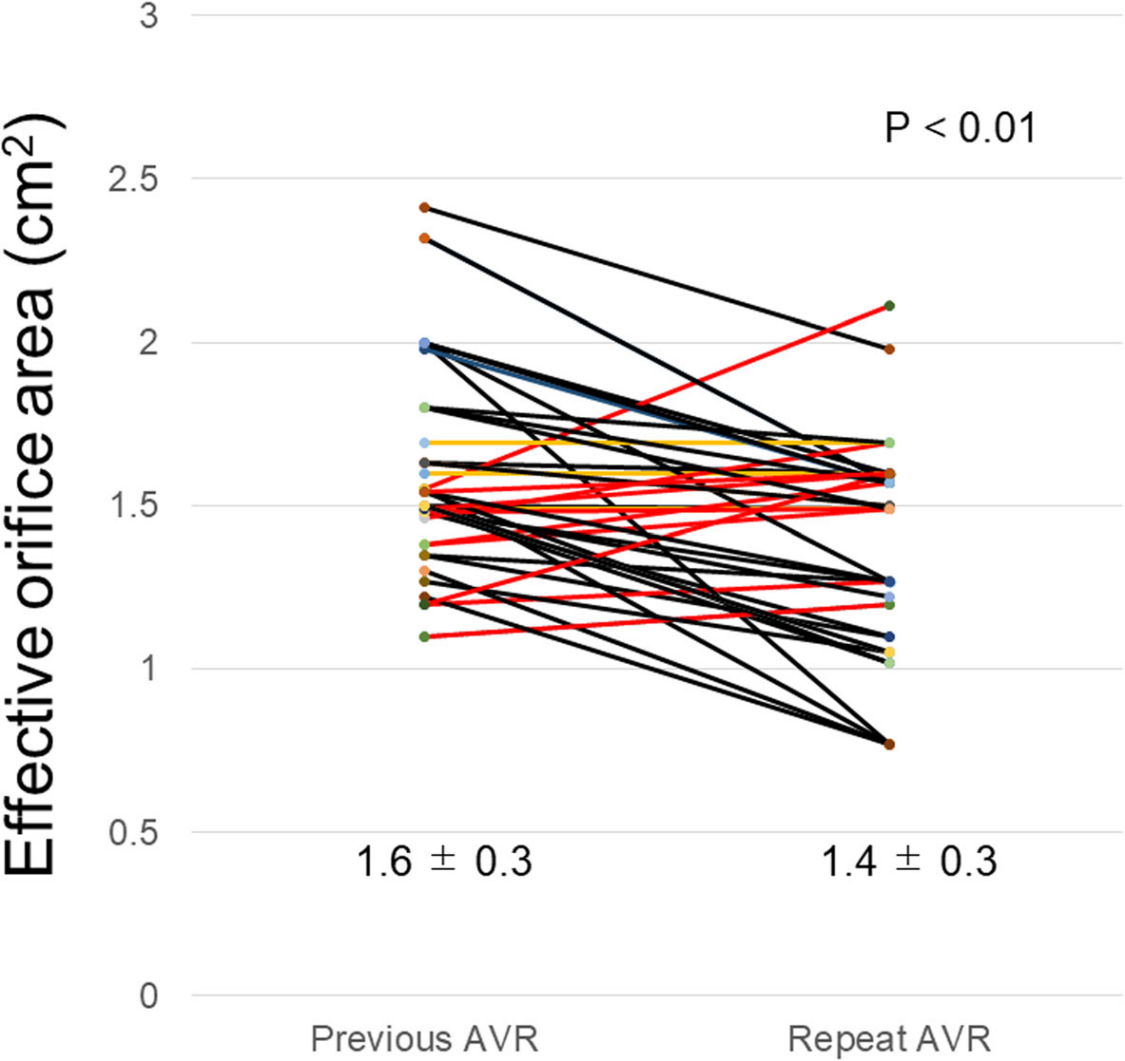
Reference EOA in repeat AVR. The reference EOA decreased in 30 cases (black line), increased in 11 cases (red line), and were unchanged in 3 cases (yellow line). AVR, aortic valve replacement; EOA, effective orifice area

The annular diameter of the new valve implanted during re-AVR was significantly smaller than that of the explanted valve that had been implanted during previous AVR (20.5 ± 2.6 vs. 22.5 ± 2.3, *p* < 0.01). The reference EOA of the new valve implanted during re-AVR was significantly smaller than that of explanted valve that had been implanted during a previous AVR (1.4 ± 0.3 vs. 1.6 ± 0.3, *p* < 0.01). The reference EOA decreased, increased and remained unchanged in 30 (68.2%), 11 (25%), and 3 (6.8%) patients, respectively (Fig. 1, Supplemental Table 1).

### Postoperative outcomes

Rates of in-hospital mortality and of perioperative complications did not significantly differ between the groups (Table 4).

**Table 4:** Postoperative outcomes

	Total (*n* = 113)	Repeat AVR (*n* = 44)	Primary AVR (*n* = 69)	*p*
Hospital death (%)	3 (2.7)	2 (4.5)	1 (1.4)	0.56
Transfusion (%)	92 (81.4)	34 (77.3)	58 (84.1)	0.46
Intubation (hours)	12 [8–22]	13 [8.25–23.5]	12 [7.25–19.5]	0.44
ICU stay (days)	3 [2–5]	3 [2–5]	3 [2–5.5]	0.88
Hospital stay (days)	18 [15–29.5]	18 [14.25–29.5]	18 [15–29.5]	0.98
Complications				
Stroke (%)	3 (2.7)	2 (4.5)	1 (1.4)	0.56
Reopen for bleeds (%)	3 (2.7)	0 (0)	3 (4.3)	0.28
Renal RT (%)	7 (6.2)	5 (11.4)	2 (2.9)	0.11
Respirator > 48 h (%)	10 (8.8)	5 (11.4)	5 (7.2)	0.51
Pneumonia (%)	11 (9.7)	6 (13.6)	5 (7.2)	0.33
Pacemaker implant (%)	3 (2.7)	2 (4.5)	1 (1.4)	0.56

*AVR* aortic valve replacement, *ICU* Intensive care unit, *RT* replacement therapy

The mean pressure gradient was greater and EOAI was smaller in the re-AVR, than the primary AVR group, whereas the incidence of moderate or severe PPM was similar between them (Table 5).

**Table 5:** Postoperative echocardiographic characteristics

	Total (*n* = 113)	Repeat AVR (*n* = 44)	Primary AVR (*n* = 69)	*p*
Mean PG (mmHg)	13.7 ± 6.3	15.2 ± 6.4	12.7 ± 6.2	0.04
Indexed EOA of PV (cm^2^/m^2^)	1.01 ± 0.33	0.92 ± 0.26	1.06 ± 0.36	0.04
Prosthesis-patient mismatch				0.87
None	72 (63.7)	27 (61.4)	45 (65.2)	
Moderate	30 (26.5)	12 (27.3)	18 (26.1)	
Severe	11 (9.7)	5 (11.4)	6 (8.7)	

*AVR* aortic valve replacement, *EOA* effective orifice areas, *PG* pressure gradient, *PV* prosthetic valve

### Survival

**Fig. 2:**
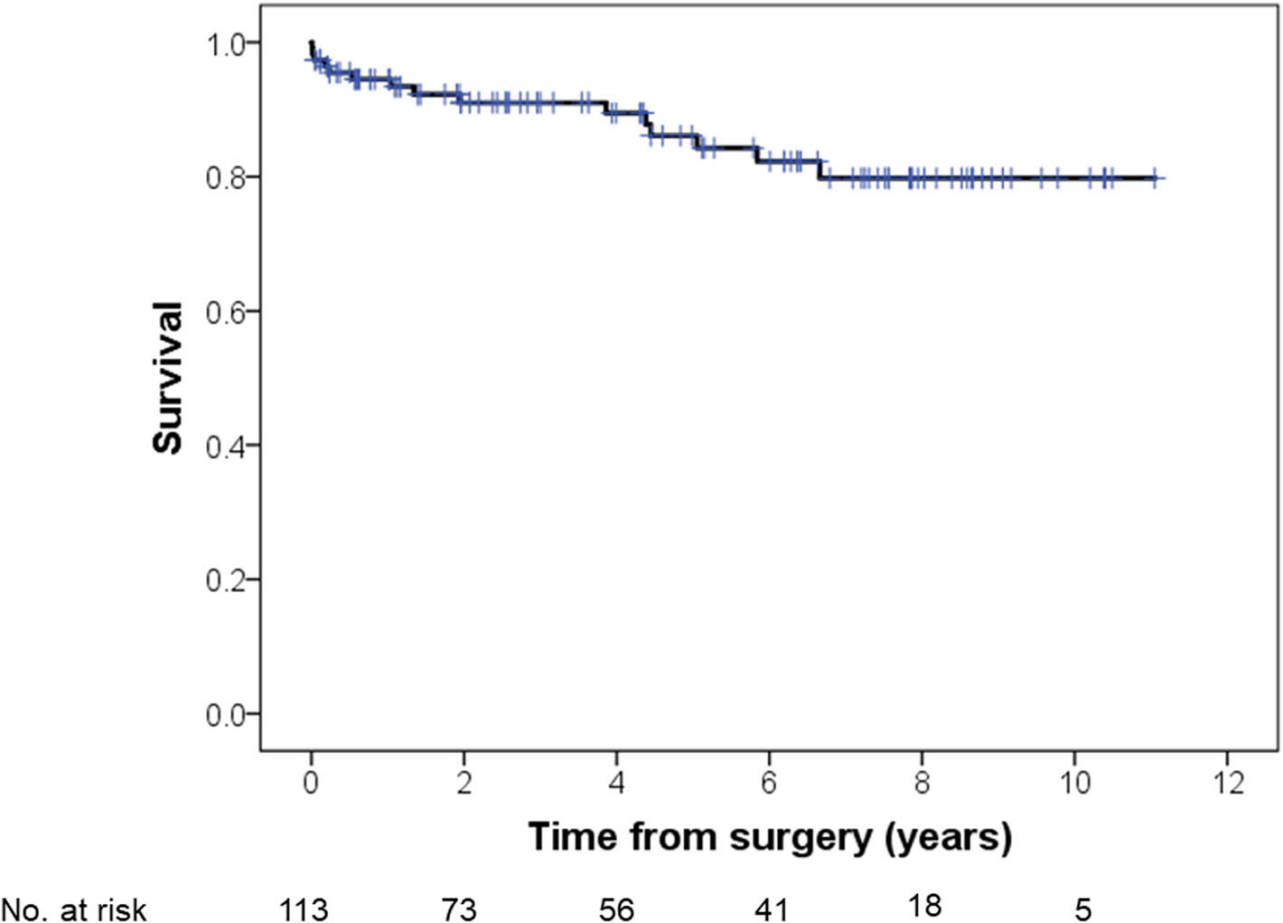
Kaplan-Meier survival of all patients who underwent AVR after any type of prior cardiac surgery. AVR, aortic valve replacement

**Fig. 3:**
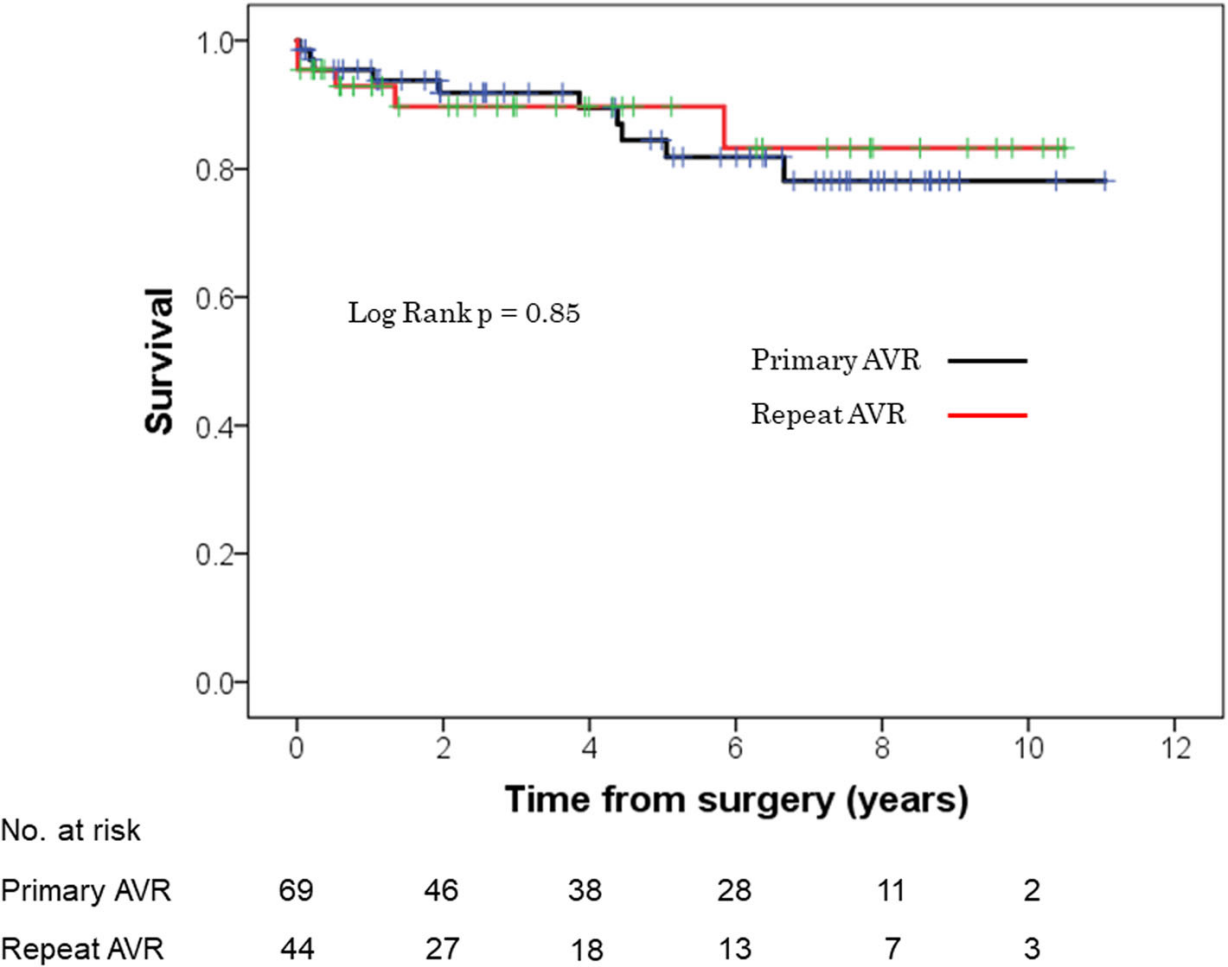
Kaplan-Meier survival of patients who underwent repeat AVR versus primary AVR after any prior cardiac surgery other than AVR. AVR, aortic valve replacement

**Fig. 4:**
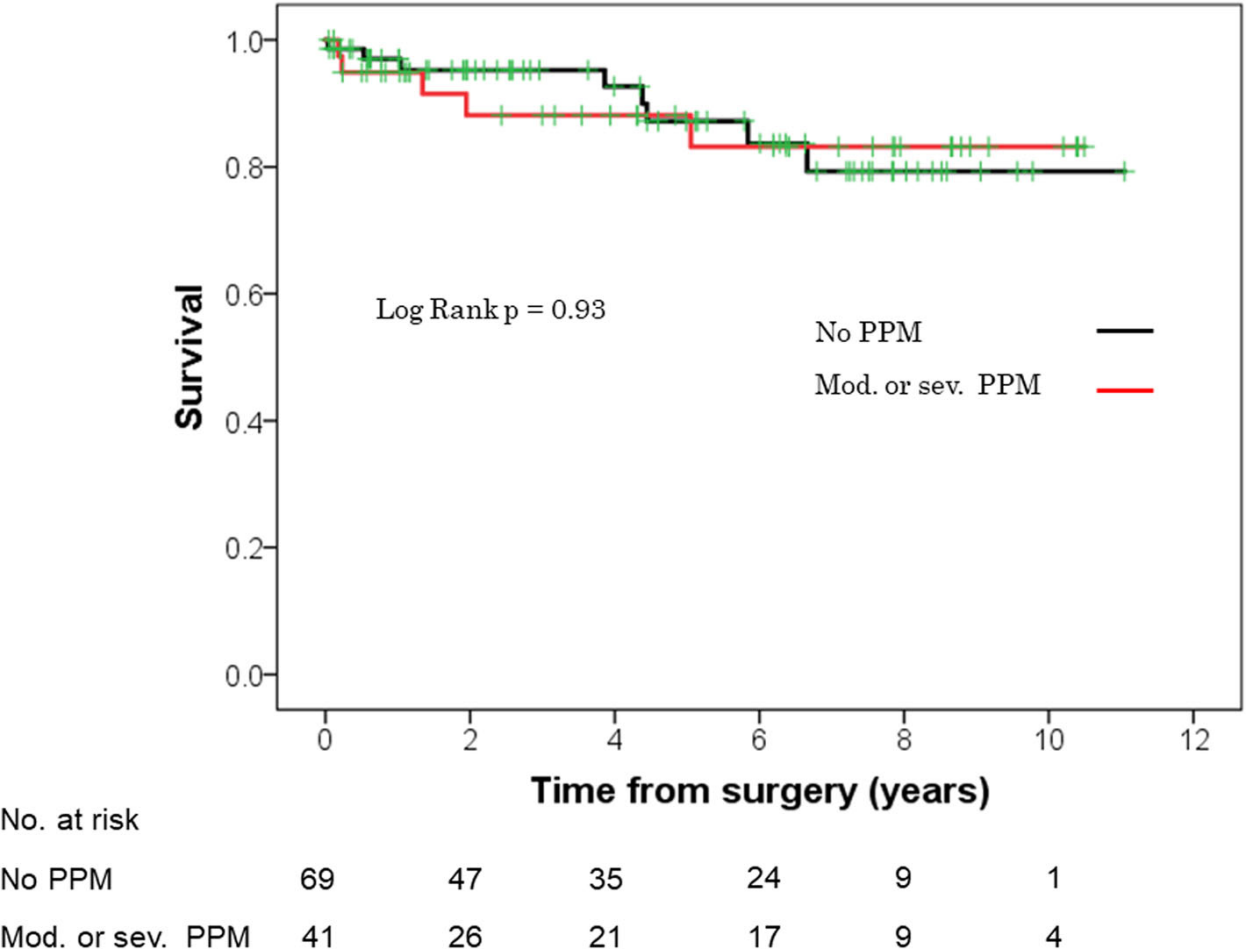
Kaplan-Meier survival of patients with or without moderate or severe PPM. PPM, prosthesis-patient mismatch

Survival estimates at 1-, 3-, and 5-year were 95, 91, and 86%, respectively overall (Fig. 2), 93, 90, and 90%, respectively, for re-AVR, and 95, 92, and 85%, respectively, for primary AVR (log-rank test, *p* = 0.85; Fig. 3). Survival rates were similar between patients with or without moderate or severe PPM (log-rank test, *p* = 0.93; Fig. 4).

## Discussion

The early and long-term outcomes of AVR after previous cardiac surgery seemed favorable and early and long-term mortality of patients after re-AVR and primary AVR did not significantly differ. The reference EOA of implanted prosthetic valves at re-AVR was smaller than that of prosthetic valves at previous AVR, the mean pressure gradient was higher, and the EOAI was lower after re-AVR than after primary AVR. However, the incidence of PPM, and the survival of patients with and without PPM were similar between the groups.

Rates of reoperative AVR after previous cardiac surgery are increasing because more patients are surviving cardiac surgery, and for longer, due to recent developments in cardiac surgery and improved postoperative management. Besides, the increasing proportion of patients who are being implanted with bioprostheses for AVR indicates that even more patients will require re-AVR. Indeed, the annual number of reoperative isolated AVR in Japan has more than tripled (from 169 to 522) between 2000 and 2016 [1, 13]. Reoperative cardiac surgery is considered high-risk, as the postoperative in-hospital mortality in Japan after isolated AVR is 7.1%, compared with 1.9% after an initial isolated AVR [1]. The overall in-hospital mortality rate for our patients was acceptable at 2.7% and re-AVR did not increase the mortality rate over that of the primary AVR.

Fallon et al. [14] reported the findings of the largest study of the incidence and long-term outcomes of PPM after AVR using The Society of Thoracic Surgeons Adult Cardiac Surgery Database. The incidence of PPM was higher in their study compared with the present findings (65% vs. 36%) and the severity of PPM was worse after reoperation. They concluded that any degree of PPM significantly decreased long-term survival.

Our findings are inconsistent with these. The incidence of PPM was notably almost double in the study of north American patients by Fallon et al. A race bias, that is, the body surface area was much larger for north American, than Japanese patients (1.9 vs. 1.5 m^2^), which might have exerted some influence.

Although the reference EOA was smaller for prosthetic valves implanted during re-AVR than during a previous AVR in the same patient, the incidence of PPM was similar between re-AVR and primary AVR in our study. The reduced EOA in re-AVR appears to be due to annular fibrosis caused by previous prosthetic valve implantation and the restriction of adherent surrounding tissue. We compared the incidence of PPM among patients with previous cardiac surgery and found some adhesions around the aortic roots of patients who underwent primary AVR. This might also explain the discrepancy between the present study and that of Fallon et al.

The survival rates of patients with and without PPM were similar in the present study. Several studies [15–17] have found that PPM do not negatively impact late survival. For this reason, we are reluctant to perform annular enlargement during AVR even when the annulus is relatively small. However, these findings like ours, were based on relatively low numbers of patients. Besides, our study included AVR with concomitant procedures in addition to isolated AVR, which could have led to insufficient statistical power.

Transcatheter aortic valve replacement (TAVR) has now become the most common procedure for patients with severe aortic valve stenosis who are considered unsuitable for surgical AVR due to excessive operative risk. Moreover, transcatheter aortic VIV implantation has recently emerged as a less invasive treatment for patients with degenerated bioprostheses. Reports have indicated that VIV is technically feasible [2–4]; thus, a clear understanding of the indications and limitations of VIV and re-AVR is now important to establish.

The PARTNER 2 Valve-in-Valve Registry [2] determined mean EOA, EOAI and pressure gradient values of 1.13 cm^2^, 0.60 cm^2^/m^2^, and 17.7 mmHg, respectively, using postoperative echocardiography, and PPM was severe in 58.4% of patients. The 30-day all-cause mortality was 2.7% and 1-year overall mortality was 12.4%. The present values (except 30-day mortality) after re-AVR were better than these.

Two meta-analyses have compared the outcomes between VIV and re-AVR for degenerated bioprostheses. Neupane et al. [18] reported that the residual mean gradient was significantly higher in the VIV group, but 30-day mortality rates did not significantly differ between the two groups. Gozdek et al. [19] reported similar results. Although re-AVR was associated with lower mean gradients and a lower incidence of PPM, neither procedural, nor 30-day mortality rates significantly differed.

The VIV approach seems a feasible option for patients with failed bioprostheses who are inoperable, or at very high risk. The gold standard should remain re-AVR, particularly for low-risk or younger patients, because it offers superior hemodynamic outcomes with low mortality rates.

Other factors favor re-AVR over VIV. Over 50% of patients in the present study required concomitant procedures including coronary revascularization, and mitral or tricuspid valve surgery. The feasibility of an additional procedure is a major advantage of surgical AVR compared with TAVR. Furthermore, VIV is not feasible for mechanical valves; the present study found that 14 mechanical valves were explanted due to pannus formation or infectious endocarditis.

Sutureless aortic valve prostheses have emerged as a useful alternative to AVR in high-risk patients. Chiariello et al. [20] described the implantation of a surgical sutureless aortic valve after removing a degenerated Mitroflow valve (Sorin, Saluggia, Italy). Early mortality and major complications did not arise, and early postoperative mean pressure gradients were low (13.1 mmHg). Santarpino et al. [21] reported the hemodynamic outcomes of reintervention for a degenerated valve that involved VIV and replacement with sutureless aortic valves. In-hospital mortality did not occur and the mean EOAI were 0.96 and 0.71 cm^2^/m^2^ in the group with sutureless valves and the group that underwent VIV. Surgical sutureless implantation seems to be more effective than VIV in terms of hemodynamic outcomes.

### Limitations

This retrospective study of a relatively small sample of a patients proceeded at a single center. Thus, we did not apply propensity matching. There was some heterogeneity between the two groups, such as previous procedure history and implanted valve type. Furthermore, the observational, univariate study design precluded definitive conclusions regarding comparisons among the groups because of selection bias and unmeasured confounders. The average body surface area in this study was relatively small because all patients were Japanese; therefore, our results may not be generalizable to Western populations. Although there was no significant difference, the primary-AVR group was slightly younger than the redo-AVR group, and the primary-AVR group had more mechanical valves. Generally, mechanical valves have a larger EOA than bioprosthetic valves; therefore, this heterogeneity might contribute to the difference in EOAI between the groups.

## Conclusions

Early and long-term outcomes of repeat AVR were acceptable and early and long-term mortality did not differ between patients who underwent re-AVR and primary AVR after prior cardiac surgery. Although the reference EOA of implanted prosthetic valve at re-AVR was smaller than that of the valve at initial AVR, re-AVR did not increase the incidence of PPM. These findings might serve as a guide for selecting re-AVR or VIV.

## Supplementary information

**Additional file 1 : Supplemental Table 1.** Valve size and EOA of explanted and implanted valves in repeat aortic valve replacement.

## Data Availability

The datasets used and/or analyzed during the current study are available from the corresponding author on reasonable request.

## Abbreviations

AVR: Aortic valve replacement
CI: Confidence interval
EOA: Effective orifice area
EOAI: Indexed EOA
PPM: Prosthesis-patient mismatch
SVD: Structural valve deterioration
TAVR: Transcatheter aortic valve replacement
VIV: Valve-in-valve
