# Supplementary material for: Incidence and influence of prosthesis-patient mismatch after reoperative aortic valve replacement: a retrospective single-center study
Source: J Cardiothorac Surg. 2020 Mar 30;15:53. doi: 10.1186/s13019-020-01094-2 (PMC7104489; doi:10.1186/s13019-020-01094-2)
Supplement: Supplementary file 1 — Additional file 1 : Supplemental Table 1. Valve size and EOA of explanted and implanted valves in repeat aortic valve replacement. [file 13019_2020_1094_MOESM1_ESM.docx]

Supplemental Table 1. Valve size and EOA of explanted and implanted valves in repeat aortic valve replacement

| Patients | Explanted valve | Valve size (mm) | TAD (mm) | EOA (cm^2)^ | Implanted valve | Valve size (mm) | TAD (mm) | EOA (cm^2^) | ΔTAD | ΔEOA |
| --- | --- | --- | --- | --- | --- | --- | --- | --- | --- | --- |
| 1 | Freestyle | 21 | 21 | 1.35 | CEP | 19 | 19 | 1.1 | -2 | -0.25 |
| 2 | Carbomedics | 23 | 23.8 | 1.63 | SJM Regent | 19 | 19.4 | 1.6 | -4.4 | -0.03 |
| 3 | SJM | 21 | 21 | 2 | SJM Regent | 19 | 19.4 | 1.6 | -1.6 | -0.4 |
| 4 | Mosaic | 23 | 19 | 1.38 | CEP Magna | 21 | 21 | 1.49 | 2 | 0.11 |
| 5 | Freestyle | 27 | 27 | 2.32 | CEP Magna | 23 | 23 | 1.57 | -4 | -0.75 |
| 6 | CEP | 23 | 23 | 1.5 | CEP Magna | 21 | 21 | 1.49 | -2 | -0.01 |
| 7 | Freestyle | 23 | 23 | 1.48 | SJM Regent | 17 | 17.4 | 1.1 | -5.6 | -0.38 |
| 8 | Carbomedics | 27 | 27.8 | 2.41 | Carbomedics | 25 | 25.8 | 1.98 | -2 | -0.43 |
| 9 | SJM | 21 | 21 | 2 | SJM Regent | 19 | 19.4 | 1.6 | -1.6 | -0.4 |
| 10 | Freestyle | 21 | 21 | 1.35 | CEP Magna | 19 | 19 | 1.27 | -2 | -0.08 |
| 11 | Mosaic | 19 | 19 | 1.2 | CEP Magna | 19 | 19 | 1.27 | 0 | 0.07 |
| 12 | HANCOCK II | 27 | 25 | 1.55 | CEP Magna | 27 | 27 | 2.11 | 2 | 0.56 |
| 13 | SJM | 21 | 21 | 2 | ATS | 18 | 18.2 | 0.77 | -2.8 | -1.23 |
| 14 | CEP | 21 | 21 | 1.3 | ATS | 18 | 18.2 | 0.77 | -2.8 | -0.53 |
| 15 | CEP Magna | 21 | 21 | 1.49 | CEP Magna ease | 21 | 21 | 1.49 | 0 | 0 |
| 16 | Bjork-Shiley | 21 | 21 | 1.55 | ATS | 18 | 18.2 | 0.77 | -2.8 | -0.78 |
| 17 | SJM | 19 | 19 | 1.6 | SJM Regent | 19 | 19.4 | 1.6 | 0.4 | 0 |
| 18 | Mosaic | 23 | 23 | 1.38 | CEP Magna ease | 23 | 23 | 1.57 | 0 | 0.19 |
| 19 | CEP Magna ease | 21 | 21 | 1.49 | ATS | 20 | 20.2 | 1.02 | -0.8 | -0.47 |
| 20 | Freestyle | 27 | 27 | 2.32 | CEP Magna ease | 23 | 23 | 1.57 | -4 | -0.75 |
| 21 | PRIMAPLUS | 21 | 21 | 1.1 | Mosaic | 19 | 19 | 1.2 | -2 | 0.1 |
| 22 | Freestyle | 23 | 23 | 1.48 | CEP Magna ease | 19 | 19 | 1.27 | -4 | -0.21 |
| 23 | Freestyle | 25 | 25 | 2 | CEP Magna ease | 19 | 19 | 1.27 | -6 | -0.73 |
| 24 | PRIMAPLUS | 21 | 21 | 1.1 | Mosaic | 19 | 19 | 1.2 | -2 | 0.1 |
| 25 | CEP Magna ease | 21 | 21 | 1.49 | Mosaic | 21 | 21 | 1.22 | 0 | -0.27 |
| 26 | Carbomedics | 23 | 23.8 | 1.63 | ATS | 22 | 22.2 | 1.5 | -1.6 | -0.13 |
| 27 | HANCOCK II | 25 | 25 | 1.46 | CEP Magna ease | 25 | 25 | 1.69 | 0 | 0.23 |
| 28 | Freestyle | 23 | 23 | 1.48 | CEP Magna ease | 21 | 21 | 1.49 | -2 | 0.01 |
| 29 | CEP Magna ease | 25 | 25 | 1.69 | CEP Magna ease | 25 | 25 | 1.69 | 0 | 0 |
| 30 | Carbomedics | 21 | 21.8 | 1.54 | ATS | 20 | 20.2 | 1.02 | -1.6 | -0.52 |
| 31 | CEP Magna ease | 21 | 21 | 1.49 | SJM Regent | 19 | 19.4 | 1.6 | -1.6 | 0.11 |
| 32 | Mosaic | 21 | 21 | 1.22 | ATS | 18 | 18.2 | 0.77 | -2.8 | -0.45 |
| 33 | Carbomedics | 23 | 23.8 | 1.63 | ATS | 22 | 22.2 | 1.5 | -1.6 | -0.13 |
| 34 | CEP Magna | 19 | 19 | 1.27 | CROWN PRT | 19 | 18.6 | 1.05 | -0.4 | -0.22 |
| 35 | Carbomedics | 25 | 25.8 | 1.98 | CEP Magna ease | 23 | 23 | 1.57 | -2.8 | -0.41 |
| 36 | Mosaic | 19 | 19 | 1.2 | SJM Regent | 19 | 19.4 | 1.6 | 0.4 | 0.4 |
| 37 | SJM | 21 | 21 | 2 | CEP Magna ease | 23 | 13 | 1.57 | -8 | -0.43 |
| 38 | PRIMAPLUS | 25 | 25 | 1.8 | CEP Magna ease | 21 | 21 | 1.49 | -4 | -0.31 |
| 39 | PRIMAPLUS | 23 | 23 | 1.5 | CROWN PRT | 19 | 18.6 | 1.05 | -4.4 | -0.45 |
| 40 | PRIMAPLUS | 23 | 23 | 1.5 | CROWN PRT | 19 | 18.6 | 1.05 | -4.4 | -0.45 |
| 41 | PRIMAPLUS | 25 | 25 | 1.8 | CEP Magna ease | 23 | 23 | 1.57 | -2 | -0.23 |
| 42 | CEP | 25 | 25 | 1.8 | CEP Magna ease | 25 | 25 | 1.69 | 0 | -0.11 |
| 43 | Carbomedics | 21 | 21.8 | 1.54 | CEP Magna ease | 19 | 19 | 1.27 | -2.8 | -0.27 |
| 44 | Carbomedics | 21 | 21.8 | 1.54 | Trifecta | 19 | 19 | 1.6 | -2.8 | 0.06 |

EOA, effective orifice areas; TAD, tissue annulus diameter
